# β subunits of voltage-gated calcium channels in cardiovascular diseases

**DOI:** 10.3389/fcvm.2023.1119729

**Published:** 2023-02-02

**Authors:** Kelvin Wei Zhern Loh, Cong Liu, Tuck Wah Soong, Zhenyu Hu

**Affiliations:** ^1^Department of Physiology, Yong Loo Lin School of Medicine, National University of Singapore, Singapore, Singapore; ^2^Cardiovascular Disease Translational Research Programme, Yong Loo Lin School of Medicine, National University of Singapore, Singapore, Singapore; ^3^NUS Graduate School for Integrative Sciences and Engineering, Singapore, Singapore; ^4^Healthy Longevity Translational Research Programme, Yong Loo Lin School of Medicine, National University of Singapore, Singapore, Singapore

**Keywords:** voltage-gated calcium channel (VGCC), Ca_V_β subunits, Ca^2+^, cardiovascular disease, post-translational modification (PTM)

## Abstract

Calcium signaling is required in bodily functions essential for survival, such as muscle contractions and neuronal communications. Of note, the voltage-gated calcium channels (VGCCs) expressed on muscle and neuronal cells, as well as some endocrine cells, are transmembrane protein complexes that allow for the selective entry of calcium ions into the cells. The α1 subunit constitutes the main pore-forming subunit that opens in response to membrane depolarization, and its biophysical functions are regulated by various auxiliary subunits–β, α2δ, and γ subunits. Within the cardiovascular system, the γ-subunit is not expressed and is therefore not discussed in this review. Because the α1 subunit is the pore-forming subunit, it is a prominent druggable target and the focus of many studies investigating potential therapeutic interventions for cardiovascular diseases. While this may be true, it should be noted that the direct inhibition of the α1 subunit may result in limited long-term cardiovascular benefits coupled with undesirable side effects, and that its expression and biophysical properties may depend largely on its auxiliary subunits. Indeed, the α2δ subunit has been reported to be essential for the membrane trafficking and expression of the α1 subunit. Furthermore, the β subunit not only prevents proteasomal degradation of the α1 subunit, but also directly modulates the biophysical properties of the α1 subunit, such as its voltage-dependent activities and open probabilities. More importantly, various isoforms of the β subunit have been found to differentially modulate the α1 subunit, and post-translational modifications of the β subunits further add to this complexity. These data suggest the possibility of the β subunit as a therapeutic target in cardiovascular diseases. However, emerging studies have reported the presence of cardiomyocyte membrane α1 subunit trafficking and expression in a β subunit-independent manner, which would undermine the efficacy of β subunit-targeting drugs. Nevertheless, a better understanding of the auxiliary β subunit would provide a more holistic approach when targeting the calcium channel complexes in treating cardiovascular diseases. Therefore, this review focuses on the post-translational modifications of the β subunit, as well as its role as an auxiliary subunit in modulating the calcium channel complexes.

## 1. Introduction

Calcium is one of the most important elements in organism, participating various physiological processes, such as heartbeat, muscle contraction, and neuronal communication (1,2). Ca^2+^ enter into the nerve, muscle, and some endocrine cells mainly through voltage-gated Ca^2+^ channels (VGCCs). Based on the membrane voltage required for activation, 10 subtypes of VGCCs were subsequently classified into high-voltage activated (HVA) and low-VA (LVA) calcium channels (3). Further studies classified Ca^2+^ currents into L- (Long-lasting), N-(Neural), P(Purkinje)/ Q-, R- (Residual), and T-(Transient) type currents, which exhibit distinct biophysical and pharmacological properties (4).

**FIGURE 1:**
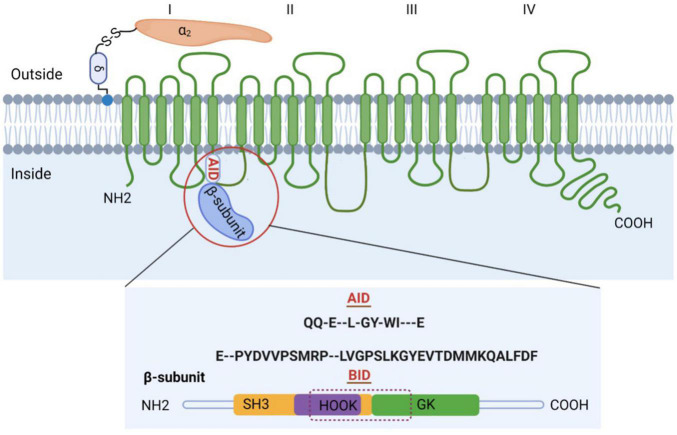
Subunit composition of high voltage-gated calcium channels (VGCCs). Schematic representation of the predicted transmembrane topology of VGCCs subunits. The complex is composed of the pore-forming subunit α_1_ (175 kDa) and two auxiliary subunits: α_2_δ and β. The AID, marked in red, is located on the I–II loop. β subunit is comprised of GK domain, Src homology 3 (SH3), HOOK region, NH2 terminus, and carboxyl terminus, with BID spanning across SH3-HOOK-GK motif. Both AID and BID have a conserved consensus motif, that is, QQ-E–L-GY-WI—E (AID) and E–PYDVVPSMRP–LVGPSLKGYEVTDMMKQALFDF (BID) (36).

The purified channel complex is composed of the pore-forming subunit α_1_ (175 kDa) and two auxiliary subunits: α_2_δ (about 160 kDa), β (54 kDa) (Figure 1) (5). The α_1_ subunit (Ca_v_α_1_) is the principal component of VGCCs and is responsible for their unique biophysical and pharmacological properties. However, proper trafficking and functions of L-, N-, P/ Q-, and R-type channels require the auxiliary subunits. In particular, β subunit is indispensable for HVA Ca_V_1 and Ca_V_2 Ca^2+^ channels. β subunit acts in many different aspects by enhancing Ca^2+^ channel currents through prevention of proteasomal degradation (6), changing the voltage dependence and kinetics of activation and inactivation, and modulating Ca_v_1 and Ca_v_2 channels by protein kinases or G protein (7).

β subunit has four subfamilies (β_1_–β_4_). β_1_ subunit is mainly expressed in brain, heart, skeletal muscle, spleen, T-cells (Table 1) (4). The β_1a_ isoform, however, is exclusively expressed in skeletal muscle where it partners with skeletal muscle Ca_v_1.1, and is irreplaceable for excitation-contraction coupling (7). β_2_ subunit is found in brain, heart, lung, nerve, nerve endings at the neuro-muscular junction, T-cells, osteoblasts (Table 1) (4). It is also the predominant β subunit in the heart, especially β_2b_ (8). β_2_ subunit knockout mice die prenatally at embryonic day 10.5 due to lack of cardiac contractions (9, 10), indicating the significant role of β_2b_ subunit in cardiovascular human diseases. More importantly, Antzelevitch et al. (11) pointed out that S481L mutation in the C-terminus of β_2b_ subunit contributed to a type of sudden death syndrome characterized by a short-QT interval and an elevated ST-segment. Cordeiro et al. (12) further found that T11l mutation in the β_2b_ N-terminus led to accelerated inactivation of cardiac L-type channels and was linked to the Brugada syndrome.

**TABLE 1:** Tissue distribution and key features of β subunits.

Subunit	Tissue distribution and key features	References
β_1_	β_1_ mainly expressed in brain, heart, skeletal muscle, spleen, and T-cells. β_1a_ isoform is exclusively expressed in skeletal muscle, partnering with skeletal muscle Ca_v_1.1, and being irreplaceable for excitation-contraction coupling.	(4, 6)
β_2_	β_2_ mainly expressed in brain, heart, lung, and nerve. β_2b_ is most abundant β subunit in the heart and is crucial for cardiac contractions.	(4, 7–9)
β_3_	β_3_ mainly expressed in brain, heart, aorta, and smooth muscle. β_3_ is associated with brain disorders, such as, memory loss, emotional disturbance, and epilepsy.	(4, 13, 14)
β_4_	β_4_ mainly expressed in brain, especially in cerebellum, kidney, and skeletal muscle. β_4_ knockout mice exhibit ataxia, seizures, absence epilepsy, and paroxysmal dyskinesia.	(4, 19)

β_3_ mostly expressed in brain, but also in heart, aorta and smooth muscle, regulating pain, memory, emotion, and even blood pressure (Table 1) (4). β_3_ subunit knockout mice showed reduced perception of inflammatory pain but not mechanically or thermally induced pain, which is probably due to reduced N-type calcium channel expression in dorsal root ganglia (13). Behaviorally, β_3_-null mice exhibit impaired working memory, lowered anxiety and increased aggression and nighttime activity (14,15). Moreover, a high-salt diet induced abnormally elevated blood pressure, reduced plasma catecholamine levels (16) and hypertrophic heart and aortic smooth muscle in β_3_ subunit knockout mice (17). These results point to a compromised sympathetic control in β_3_ subunit knockout mice, likely due to reduced N- and L-type channel activity (18). A recent study comparing epileptic patients with non-epileptic individuals identified three mutations in β_3_ subunit that were present only in patients (19), which further associated β_3_ subunit with brain disorders.

β_4_ subunit expressed in cerebellum, kidney, and skeletal muscle, but not in the cardiac muscle of adults (Table 1) (4). β_4_ subunit knockout mice, which was generated by an insertion that causes exon skipping and a premature stop codon in the gene for β_4_ subunit, exhibited ataxia, seizures, absence epilepsy, and paroxysmal dyskinesia (20). This could be caused by a 50% up-regulation of T-type Ca^2+^ channels in thalamic neurons (21). Recent studies have, respectively, indicated that lethargic mice also show impairment of parallel fiber volley and Purkinje cell firing in cerebellum (22) and a modified electro-oculogram (23). More importantly, three mutations in the gene encoding of β_4_, that is, R468Q, R482X, and C104F, were identified in patients with seizures and epilepsy (24, 25).

Overall, β subunit is crucial not only in the protein expression level of calcium channels, but also in the channel activation and inactivation. Various heterologous expression systems with all four subfamilies of β subunit and all Ca_V_1 and Ca_V_2 subunits have shown that β subunit can function as a chaperone to dramatically increase the surface expression of Ca_V_1 and Ca_V_2 channels (26). Moreover, β_1_ and β_2_ knockout mice have severely reduced Ca^2+^ currents in heart, while overexpression of β subunit using adenoviruses can increase Ca^2+^ channel current density in native cardiac cells (27,28). β subunit can also prevent proteasomal degradation of calcium channels induced by increased ubiquitination *via* K48-linked ubiquitin chain, which was caused by its association with ret finger protein 2 (RFP2), an ER-localized RING finger E3 ligase (6,29). In addition, studies on β subunit knockout mice have shown that β subunit can shift the voltage dependence of activation to more hyperpolarized voltages by 10–15 mV (10). This shift is also visible at the single-channel level, as indicated by Luvisetto et al. (30). Furthermore, β subunit also modulates voltage-dependent inactivation, which reduces the amount of Ca^2+^ entering the cell following depolarization and decreases the number of channels responsible to subsequent depolarizations, causing a shift to more depolarized voltages by 10–20 mV (8, 31).

## 2. Crystal structure of β subunit

Previous studies have revealed that β subunit is comprised of five distinct regions, namely, guanylate kinase (GK) domain, Src homology 3 (SH3), HOOK region, NH2 terminus, and β-interaction domain (BID), based on amino acid sequence alignment, biochemical and functional studies, and molecular modeling (8,32). The GK domain, SH3 domain, and HOOK region constitute the core of β subunit. GKs can catalyze the reversible phosphoryl transfer from ATP to guanosine monophosphate (GMP) to produce ADP and GDP. SH3 domain contains a well-preserved PxxP motif-binding site and thus has the potential to bind PxxP motif-containing proteins. Therefore, the GK domain and SH3 domain are protein interaction modules in β subunit, engaging in protein-protein interactions (33).

With the development of crystallography, the crystal structure of the β subunit core was reported in 2004, verifying that β core indeed contains an SH3 domain and a GK domain, linked by HOOK region (34–36). Crystal structures of yeast GKs show that these enzymes have a catalytic site harboring the GMP- and ATP-binding pockets (37), which further explains its catalytic ability. The SH3 domain has a similar folding as canonical SH3 domains do. However, its last two β sheets are non-continuous, separated by the HOOK region (34–36). The HOOK region is variable in length and amino acid sequence among the β subfamilies. In the crystal structures, a large portion of the HOOK is unresolved due to poor electron density, indicating that it has a high degree of flexibility (34–36). A recent study also revealed the NMR structure of the NH_2_ terminus, which consists of two α-helices and two antiparallel β sheets (38). One of the two α-helices in the NMR structure is equivalent to the very first α-helix in the β core structures. Superposition of this helix in the two structures reveals that the NH_2_ terminus is oriented away from the core.

Crystal structure of the β core not only verified the component of β core, but also showed that SH3 and GK domains interact intramolecularly (34–36). The interaction is strong enough such that hemi-β fragments containing the NT-SH3_βstrand__1–4_-HOOK module and the SH_3βstrand__5_-GK-CT module can associate biochemically *in vitro* and reconstitute the functionality of full-length β subunits when they are coexpressed in cells (39–41). The strong intramolecular SH3-GK interaction comes from the last β sheet of β-SH3 (SH3_βstrand__5_), which is directly connected to the GK domain, and interacts extensively with both the GK domain and the rest of the SH3 domain, strengthening the otherwise weak interactions at the SH3-GK interface (42). Apart from the interaction between SH3 and GK domains within β subunit, Pragnell et al. (32) also found that β subunit binds to α-interaction domain (AID) domain within α_1_ subunit, a high-affinity site located in the cytoplasmic loop connecting the first two homologous repeats of α_1_ subunit. AID is comprised of 18 residues, with a conserved consensus motif (QQxExxLxGYxxWIxxxE) in all Ca_V_1 and Ca_V_2 channels. The AID binds to all four β subunits through the BID, a 31-amino acid segment of β subunit (43). Single mutations in either AID or BID can greatly weaken the α_1_–β interaction, as indicated by *in vitro* binding experiments (44).

## 3. Post-translational modifications of β subunit

The calcium channel β subunits are known to be regulated by various post-translational modifications, namely, phosphorylation and palmitoylation. Pioneering studies from the 1980s revealed the rapid phosphorylation of the RRPTP motif of the β_1a_ subunit by protein kinase A (PKA) and cAMP-dependent kinases within skeletal muscles (45). Notably, in cardiomyocytes, cAMP-dependent phosphorylation was later identified to be the primary phosphorylation of the β subunits (46). As such, phosphorylation of the β subunits was thought to be a signal transduction pathway for the up-regulation of L-type calcium channels.

### 3.1. Phosphorylation

β_2a_, the main β subunit within the cardiac milieu, was empirically reported to be phosphorylated by PKA on three serine residues–Ser459, Ser 478, and Ser479 of the C-terminus (47). Interestingly, these phosphorylation sites were not conserved within the other β subunit isoforms. This therefore raises the question for whether phosphorylation of the β_2a_ subunit is indeed important for its function. However, an overexpression paradigm in cardiomyocytes revealed no significant modulation of the Ca_V_1.2 channels by the β_2a_ subunit (48). In that study, it was observed that a phosphorylation-deficient β_2a_ subunit mutant equally up-regulated the functions of Ca_V_1.2 channels in heart cells. This result suggests that the phosphorylation of the β_2a_ subunits in heart cells may be involved in the facilitation of other signaling pathways rather than modulating Ca_V_1.2 channel functions. Nevertheless, it should be noted that the presence of the β_2a_ subunit is still important for increasing the open probability (*P*_o_) of the α_1_ subunit even when compared to the other β subunit isoforms (8).

In a more recent study, the use of liquid chromatography-mass spectrometry (LC-MS/MS) elucidated the *in vivo* phosphorylation of two residues of the β_1a_–Ser193 and Thr205 (49). Both residues are located within the HOOK domain of the β_1a_ subunit, and, interestingly, are conserved across the various β subunit isoforms. Although *in silico* prediction models suggested the possible phosphorylation of Ser193 of the β_1a_ by casein kinase II, *in vitro* experiments proved otherwise, however, on the other hand, Thr205 of the β_1a_ was observed to be phosphorylated by PKA. Functionally, electrophysiological whole-cell patch clamp techniques revealed the phosphor-modulatory functions of the two residues. Through the site-directed substitutions of the conserved phosphorylated residues, S152E phosphomimetic of the conserved Ser residue within β_2b_ showed a significantly decrease in the peak current density of Ca_V_1.2 channels. Moreover, the voltage-dependent activation and inactivation of Ca_V_1.2 channels were right-shifted toward more positive potentials. As expected, S152A phospho inhibitory substitution showed opposing effects. Conversely, it is interesting to note that neither the phosphomimetic nor phospho inhibitory substitution of T164D and T164A of β_2b_, respectively, revealed no observable differences in either Ca_V_1.2 peak currents or its voltage-dependent activities. However, it was noted that calcium-dependent inactivation of β_2b_^T164D^ was significantly increased but not in β_2a_^T164D^, thus suggesting a subunit-specific effect. It may be possible that the differential effect arose from other modifications of the β_2a_ subunit, such as palmitoylation. Nevertheless, it is clear that phosphorylation of either the conserved Ser or Thr residues within the HOOK domain of β subunits allowed for the modulation of the α_1_ channel properties.

Even more recently, a β subunit-dependent modulation of Ca_V_1.2 channels by Rad protein was reported (50–52). Rad is a member of the Ras/GTPase superfamily and was characterized for its unique GTPase-activating protein (GAP)-like activity (53). It was reported that substitution of specific residues into alanine in Rad (Arg208 and Leu235) or β_2a_ subunit (Asp244, Asp320, and Asp322) abolished the interaction between these two proteins (54). Furthermore, these mutations also attenuated the hyperpolarizing shift in the current-voltage curve of Ca_V_1.2 channels, thereby leading the authors to conclude that the interaction between Rad and β_2a_ is necessary for the cAMP-PKA regulation of Ca_V_1.2 channels. Moreover, using Förster resonance energy transfer (FRET), the authors further showed the robust interaction between Rad and β_2a_, which complements the reports from earlier studies (50,55). Here, the coexpression of only the PKA catalytic subunit with Rad abolished the interaction between Rad and β_2a_. It was also reported that similar results were observed between phosphorylated Rad and the other β subunit isoforms. Therefore, taken together, these data strongly suggest that the phosphorylation of Rad functions to inhibit its interaction with the β subunits. Further studies showed that Rad-phosphosite-mutant mice (4SA-Rad) displayed weakened adrenergic activation of calcium channels, which thus produced profound physiological effects: reduced heart rate with increased pauses, reduced basal contractility, attenuated of β-adrenergic contractile response, and diminished exercise capacity (52).

In addition to PKA, protein kinase C (PKC) has also been identified to phosphorylate the β subunit (56). Here, proline and serine residues within the BID were phosphorylated, and are located between the SH3 and GK domains (43). Similarly, phosphomimetic substitutions of phosphorylated residues were made and the function of the resultant phosphomimetic β_1b_ subunit mutants were tested. In their study using *Xenopus* oocyte cells, peak current density of the α_1_ subunit was observed to be increased by P221R but decreased by S228R when compared to wild-type β_1b_ subunit. Both P221R and S228R decreased voltage-dependent activation of the α_1_ channels. On the other hand, while S237R did not affect α_1_ current density, S237R increased voltage-dependent activation of the α_1_ channels. Biochemical results revealed that P221R and S228R mutants were able to interact with the α_1_ subunit similar to that of the wild-type β_1b_ subunit. However, S237R completely abolished any interactions with the α_1_ subunit. Therefore, these results suggest that PKC phosphorylation of residues within the BID serves to regulate specific functions of the β subunit, thereby modulating the α_1_ channel properties accordingly.

### 3.2. Palmitoylation

As aforementioned, the β_2a_ subunit, unlike the other β subunit isoforms, is also regulated by palmitoylation, which describes the process of covalent attachment of fatty acid chains to cysteine residues *via* a thioester linker (57–59). Palmitoylation of the β_2a_ subunit has been reported in various cell lines such as HEK 293 and Sf9 insect cells and was later identified to occur on Cys3 and Cys4 of the N-terminus. Functionally, confocal imaging revealed that palmitoylation of the β_2a_ subunit was important for regulating the localization of the protein toward the plasma membrane (57). In contrast, palmitoylation-deficient β_2a_ subunits remained largely punctate and intracellular. Moreover, through whole-cell patch clamp experiments, it was observed that the *I*_Ca,L_ of Ca_V_1.2 channels was much less per amount of charge movement when co-expressed with a palmitoylation-deficient β_2a_ subunit. Palmitoylated wild-type β_2a_ subunits also slowed inactivation of the channel complex. However, it should still be noted that even in the absence of palmitoylation, the β_2a_ mutant was still able to facilitate Ca_V_1.2 surface membrane trafficking, presumably through preventing Ca_V_1.2 turnover *via* ERAD (6).

### 3.3. S-nitrosylation

β subunits are also modified by S-nitrosylation, a reversible post-translational modification of proteins whereby cysteines are S-nitrosylated at sulfhydryl groups (60). β subunit S-nitrosylation was reported to be required for nitric oxide-mediated Ca_V_2.2 down-regulation in a subtype-dependent manner as β_1_ or β_3_ subunits resulted in stronger reduction in Ca^2+^ currents compared with β_2_ or β_4_ subunits (61). Moreover, Cys346Ala substitution of β_3_ subunit significantly ablated the inhibitory effect of S-nitroso-N-acetylpenicillamine (SNAP), a nitric oxide donor, on Ca_V_2.2 channel function (61). Therefore, while not expressed within the cardiovascular system, N-type calcium channels are important for proper neurological function and S-nitrosylation is currently being investigated for pain treatment, whereby Ca_V_2.2 channels have been reported to be important for the modulation of pain (62). However, the molecular mechanisms by which β subunit S-nitrosylation affects calcium channel function remain to be investigated. In contrast, a recent study on Ca_V_1.2 channel reported that β subunit is not involved in the nitric oxide-mediated down-regulation of Ca_V_1.2 channels (63) as the β subunits-free S-nitrosylated Ca_V_1.2 channels were still able to degraded at lysosome, a novel mechanism underlying nitric oxide-induced vasodilation. It is noteworthy that β_3_ subunit mutant carrying Cys346Ala substitution could be used in the Ca_V_1.2 study to further confirm the roles of β subunit S-nitrosylation in Ca_V_1.2 down-regulation by nitric oxide.

## 4. Roles of β subunit in calcium channel trafficking

**FIGURE 2:**
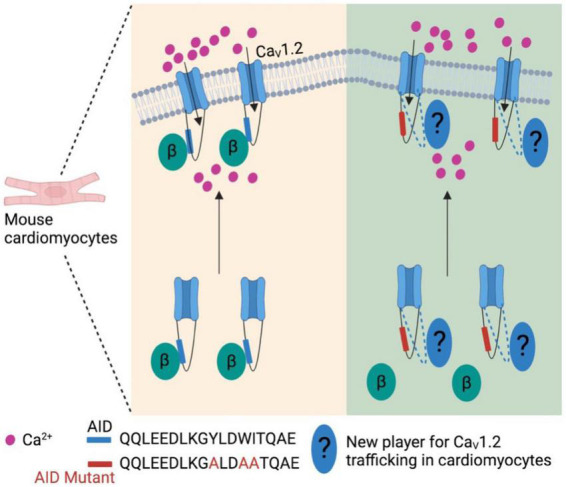
β subunit-less Ca_V_1.2 channels are still trafficked to cell surface in mouse cardiomyocytes. At least in mouse cardiomyocytes β subunit is required for β-adrenergic stimulation on Ca_V_1.2 channels, but not trafficking to plasma membrane. This indicates new player(s) is involved in the trafficking of cardiac Ca_V_1.2 channels.

More recently, the generally accepted notion that the β subunit is integral for membrane trafficking of α_1_ subunits is now brought back into discussion. In cardiomyocytes expressing transgenic α_1C_ mutants that is unable to bind β subunits, α_1C_ subunits were still observed to be trafficked to the sarcolemma *in vivo*, and was also able to sustain normal excitation-contraction coupling (Figure 2) (64). However, these α_1C_ mutants were not stimulated by agonists of the β-adrenergic pathway, thereby showing significant impairment of β-adrenergic stimulation of contractility. Taken together, these data suggest that while the β subunit is still integral for modulating channel biophysical properties, it may be dispensable in the context of maintaining proper α_1_ subunit membrane trafficking and basal electrophysiological functions in specific cell types. Nevertheless, further work needs to be done to validate this hypothesis as well as identify potential targets that may replace β subunit for the α_1_ subunit membrane trafficking in cardiomyocytes.

## 5. β subunits in cardiovascular diseases

β subunits play important roles in heart and vessels based on their dramatic effects on L-type calcium channels. Cardiac−specific β_2a_ overexpression was reported to induce cardiac hypertrophy with reduced ejection fraction in 6-month-old mice. This could be due to the increased Ca^2+^ influx through Ca_V_1.2 channels and activated hypertrophic Calcineurin-NFAT3 (nuclear factor of activated T cells) and CaMKII/HADC5 (calcium/calmodulin−dependent protein kinase II/histone deacetylase 5) signaling pathways in β_2a_-transgenic mice (65). Moreover, another study also reported that the same β_2a_−transgenic line displayed more severe cardiac hypertrophy under phenylephrine stimulation, while non-phosphorylated mutant β_2a_-overexpressing mice showed weakened responses to phenylephrine-induced cardiac hypertrophy. This study revealed that CaMKII−mediated phosphorylation of β_2a_ subunits in caveolae is essential for cardiac dysfunction induced by chronic α_1_-adrenergic stimulation (66). These results are in line with the study that reduction in Ca^2+^ influx through Ca_V_1.2 channels by short hairpin-mediated knockdown of β_2_ subunits attenuated the cardiac hypertrophy-induced by pressure overload in mice (67).

**FIGURE 3:**
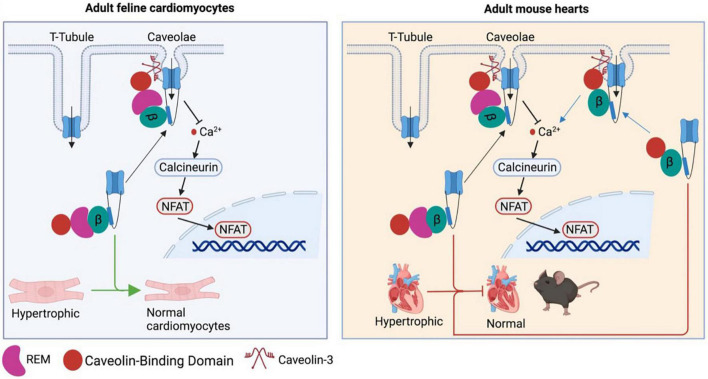
Controversy over the caveolae-resident Ca_V_1.2 calcium channel as the hypertrophic Ca^2+^ source. CBD-REM generated by a Ca_V_1.2-inhibitory domain of REM fused to a caveolin–binding domain is able to significantly inhibit the hypertrophic signaling by blocking the NFAT translocation to nucleus in adult feline left ventricular cardiomyocytes, while selective overexpression CBD-REM or CBD-CBD–β_2a_ in cardiac muscles fail to alter cardiac function of mice subjected to pressure overload-induced cardiac dysfunction.

Although β subunits are involved in the pathogenesis of cardiac hypertrophy, it remains unclear how β subunits regulate Ca_V_1.2 channel function in cardiomyocytes as Ca_V_1.2 channels were localized at two different microdomains in ventricular and atrial cardiomyocytes: T−tubule and caveolae (68–71). Moreover, there has been a debate on where is the source of hypertrophic Ca^2+^. A Ca_V_1.2-inhibitory domain of REM, a member of the Rad, Rem, Rem2, and Gem/Kir (RGK) GTPase family that is known to potently inhibit Ca_V_1.2 channel function (72), was fused to a caveolin−binding domain to generate the chimeric protein Caveolin-binding domain (CBD)-REM. CBD-REM was shown to inhibit the NFAT translocation to nucleus in adult feline left ventricular myocytes, although it had no effects on total Ca^2+^ current density (Figure 3) (73). This work indicates that Ca_V_1.2 channels from caveolae could be the pathway for hypertrophic Ca^2+^, while T-tubule-resident Ca_V_1.2 channels mainly account for contractile Ca^2+^. In contrast, with cardiac-specific overexpression of CBD−REM or a caveolae−targeted Ca_V_1.2 activator (CBD−β_2a_, a mutated β_2a_^C3S/C4S^ fused C−terminal to CBD) in mice, both transgenic mice with pressure overload-induced cardiac hypertrophy did not display significant changes in cardiac function compared to wild-type mice (Figure 3) although CBD−β_2a_ potentiated the NFAT translocation in feline cardiomyocytes (74).

The mechanisms underlying the differential effects of CBD−β_2a_ in mouse heart and feline ventricular myocyte remain to be investigated. As L-type calcium channels were reported to be redistributed to caveolae from T-tubule in failing human and rat cardiomyocytes, which led to increased open probability of Ca_V_1.2 channels and early afterdepolarization (75), one could expect that the effect of CBD−β_2a_ on caveolae-resident Ca_V_1.2 channels may be weakened by increased Ca_V_1.2 channel numbers in caveolae of failing mouse cardiomyocytes. In addition, it is noteworthy that caveolin-3 was reported to bind to β_2c_ and β_2a_ only, not β_2b_, β_2d_, β_3_, β_4_ in mouse ventricular myocytes, and a Caveolin-3^P104L^ mutant overexpressed in neonatal mouse cardiomyocytes remarkedly reduced the β_2c_ trafficking to cell surface through Caveolin-3 retention in Golgi complex (76). This finding raises a concern that CBD−β_2a_ may not be able to bring sufficient Ca_V_1.2 channels to caveolae in cardiomyocytes. More importantly, as mentioned above in this review β subunit-less Ca_V_1.2 channels are still able to be trafficked to cell surface in mouse ventricular myocytes (64), which indicates that overexpression of CBD−β_2a_ may not produce significant effects on Ca_V_1.2 levels at plasma membrane.

In addition to heart diseases, β subunits also have essential roles in vascular diseases. It has been reported that both total Ca_V_1.2 channel level in aortas and L-type Ca^2+^ current in isolated aortic smooth muscle cells were reduced in β_3_^–/–^ mice, although the basal systolic blood pressure remained normal (16, 17, 77). However, compared to wild-type mice, β_3_^–/–^ mice developed significantly higher systolic and diastolic blood pressure when fed with high salt diet (17) or subcutaneously infused with Angiotensin II for 2 weeks (77). Why global loss of β_3_ does not induce the phenotype of hypertension? It could be due to the abundant expression of β_3_ subunits in multiple organs as mentioned in section “1. Introduction.” β_3_^–/–^ mice have been found to exhibit enhanced performance in several memory tasks through increased N-Methyl-D-Aspartate receptor (NMDAR) currents and NMDAR-dependent long-term potentiation (14), suggesting that β_3_ loss is able to activate NMDAR. Intriguingly, NMDA receptor activation induces renal vasodilation by increasing the epithelial sodium channel-dependent connecting tubule-glomerular feedback responses (78). These results suggest lack of gross phenotype in hypertension in β_3_^–/–^ mice could be due to the compensatory effects from activation of NMDAR in kidney. A smooth muscle-specific knockout mouse of β_3_ subunits may more precisely validate the roles of β_3_ subunit in blood pressure regulation.

## 6. Potential β subunits-targeted therapeutic development

Given that β subunits play key roles in regulating the function of L-type calcium channels, the major calcium channels in cardiovascular systems (29, 79), thus β subunits have been considered as targets for developing new therapeutics of cardiovascular diseases.

**FIGURE 4:**
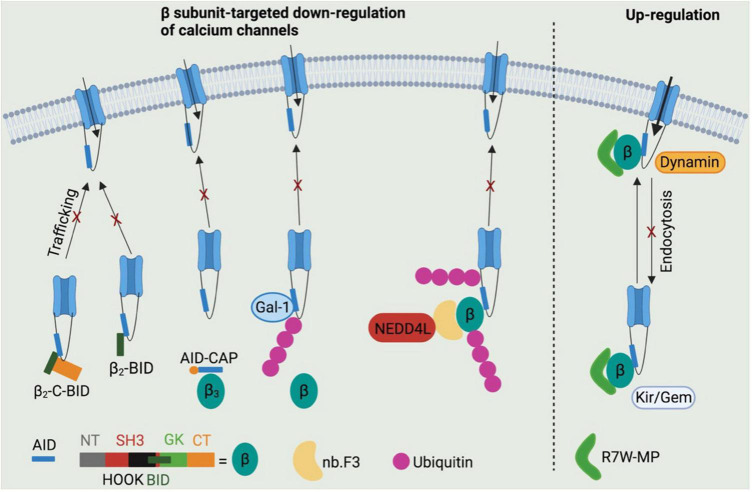
Potential β subunits-targeted therapeutic development through modulating calcium channel functions. β_2_-C-BID and β_2_-BID, lacking for the channel trafficking-required motifs, occupy the β subunits-binding sites within AID domain, thereby leading to reduced trafficking of channels to cell surface. Stapled AID peptides disrupt the interactions between calcium channels and β_3_ subunits, but not β_2_ subunits, which also results in down-regulation of calcium channel function. Galectin-1 is able to displace β subunits from calcium channels by binding to exon 9 C-terminus and thus to inhibit channel function by exposing lysines within I–II loops to ubiquitination. A β subunit-binding nanobody nb.F3 fused with catalytic domain of NEDD4L E3 ligase significantly blocks the calcium channel function by strongly enhancing the ubiquitination level. R7W-MP peptide, which binds to tail-binding domain (TID) domain within the β_2_ subunit Src homology 3 (SH3) domain, is able to up-regulate calcium channel function *via* multiple mechanisms, such as preventing channel degradation *via* ubiquitin-protease system, inhibiting the channel endocytosis by displacing dynamin from β subunits and facilitating the channel trafficking to cell surface by displacing Kir/Gem from β subunits.

Two truncated N-terminus-less β_2_ subunit including the BID and C-terminus (β_2_-C-BID) or BID only (β_2_-BID) have been reported to bind to Ca_V_1.2 channels intracellularly, while lacking the motifs required to target the Ca_V_1.2 channel complex to cell surface (80). Both β_2_-C-BID and β_2_-BID displayed strong dominant-negative effects on L-type Ca^2+^ current density in HL-1 cells as shown in Figure 4 (80), a cardiac muscle cell line derived from AT-1 mouse atrial cardiomyocyte tumor lineage (81). These β_2_ subunit decoys represent potential therapeutics to reduce Ca_V_1.2 surface expression in cardiovascular pathologies featured by up-regulation of Ca_V_1.2 channels. However, the ability of β_2_-C-BID and β_2_-BID to interfere with the protein interactions between L-type calcium channels and β_2_ subunits and to reduce total and surface Ca_V_1.2 channels remains to be tested.

In addition to BID domain, α_1_-ID within I–II loop of L-type calcium channels is also considered a target to regulate the protein interactions between β subunits and calcium channels. An AID peptide with *meta-xylyl* (m-xylyl) staple incorporated at N-terminal (AID-CAP) was designed to enhanced helical content that bind to β subunits in a native-like manner (82). AID-CAP was shown to significantly reduce the peak Ca^2+^ current in *Xenopus* oocytes co-expressing β_3_ subunits and wild-type Ca_V_1.2 channels, or β_2_ subunits and mutant Ca_V_1.2^Y437A^ channels carrying a mutation within AID that weakens the β-binding affinity by about 1,000-fold (Figure 4) (83). In contrast, AID-CAP failed to alter the Ca^2+^ current in *Xenopus* oocytes expressing β_2_ subunits and wild-type Ca_V_1.2 channels, suggesting that Ca_V_1.2-β_2a_ protein complexes stably resist kinetic competition by injected AID-CAP peptides. This could be due to the similar binding affinity of wild-type Ca_V_1.2 channels or AID-CAP to β_2_ subunits (82) or palmitoylation mediated β_2a_ subunit anchoring to the membrane (84). Overall, the stapled AID peptides show strong binding affinity to Ca_V_1.2 channels with negatively regulatory effects in β isoform-selective manner. However, further studies need to conducted to validate their effects of Ca_V_1.2 protein levels and the Ca_V_1.2-β protein interactions by biochemical assays and to test their roles in cardiovascular diseases using multiple rodent models, such as roles in hypertension using spontaneously hypertensive rats.

Regarding the Ca_V_1.2-β protein interactions, Galectin-1, a member of β-galactoside-binding protein family (85), has been identified as a new Ca_V_1.2-binding partner that interact with I–II loop in a splice isoform-specific and Ca_V_1.2 channel-selective manner (86), and has emerged as a novel target for hypertension treatment through disrupting Ca_V_1.2-β protein interaction and promoting the proteasomal degradation of Ca_V_1.2 channels (87). Galectin-1 was reported to bind to residues Asp457 and Glu459 within exon 9*-null I–II loop of Ca_V_1.2 channels, thereby removing β subunits from Ca_V_1.2 channels and masking the endoplasmic reticulum export signals within exon 9 fragment. β subunit displacement further exposed lysines within I–II loop to poly ubiquitination, which increases the proteasomal degradation of Ca_V_1.2 channels (Figure 4) (87). More importantly, the negative regulation on Ca_V_1.2 channels function by Galectin-1 was found to effectively and stably control hypertension in spontaneously hypertensive rats when Galectin-1 was overexpressed in smooth muscles using adeno-associated virus as a vector (87). It is noteworthy that the Ca_V_1.2-binding sites within Galectin-1, which may help to develop anti-hypertensive Galectin-1-based peptides, have not been identified.

Recently an immunized llama nanobody nb.F3 has been isolated and found to interact with β_2b_ with a high binding affinity (*K*_d_ = 13.2 ± 7.2 nM) (88). The nanobody itself has no effects on calcium channel function. Intriguingly, when the C-terminus of nb.F3 was fused to the catalytic Homologous to the E6-AP Carobxyl Terminal (HECT) domain of NEDD4L, an E3 ubiquitin ligase (89), the resulted construct, named Ca_V_-aβlator, completely blocked the Ca^2+^ current from various high VGCCs overexpressed in HEK 293 cells, and from endogenous calcium channels in guinea pig ventricular cardiomyocytes, murine dorsal root ganglion neurons and pancreatic β cells (Figure 4) (88). This study proposed a potent genetically encoded general inhibitor for β subunit-binding calcium channels, which, however, remains to be validated in disease models.

In addition to β subunits-targeted negative modulation of calcium channel function, a peptide R7W-MP containing an oligoarginine (R7W) cell-penetrating peptide and a fragment of β_2_ subunit C-terminal coiled-coil tail was designed to stabilize Ca_V_1.2 channels. Akt-phosphorylated β_2_ subunit C-terminal tail was reported to bind to the tail-binding domain (TID) within β_2_ SH3 domain, which induced a structural rearrangement of β_2_ subunit and thereby stabilized Ca_V_1.2 channels by preventing the proteasomal degradation (90,91). R7W-MP peptide, by targeting TID domain within the β_2_ subunit SH3 domain, was able to prevent Dynamin from binding to β_2_ subunit SH3 domain, thereby protecting Ca_V_1.2 channels against endocytosis (91,92). Moreover, R7W-MP peptide also facilitated Ca_V_1.2 chaperoning to the plasma membrane by preventing the interaction between β_2_ subunits and Kir/Gem (Figure 4) (91), a member of the RGK small GTP-binding protein family reported to decrease the level of L-type calcium channels at cell surface (93, 94). More importantly, R7W-MP restores cardiac function in a diabetic cardiomyopathy mouse model through increasing Ca_V_1.2 current density.

## 7. Conclusion

β subunits have been widely studied in various cardiovascular disease conditions and plays important roles in cardiac hypertrophy, diabetic cardiomyopathy, and hypertension, although some controversies remain. More strategies modulating high VGCCs have been developed by selectively targeting β subunit itself or the protein interactions between calcium channels and β subunits. Compared to calcium channel blockers to completely ablate the activity of L-type calcium channels, it may be advantageous to partially reduce Ca^2+^ influx by inhibiting the up-regulation of Ca_V_1.2 channels in cardiovascular diseases, such as hypertension. These findings provide proof-of-principle for this concept by showing that targeting β subunits could normalize Ca_V_1.2 channel expression, which may be used as new targets for therapeutics of cardiovascular diseases.

## Author contributions

ZH, KL, and CL outlined, drafted, and contributed to the writing of the manuscript. TS critically edited and finalized the manuscript. All authors approved the final version of the manuscript.
